# Retinoprotective Effects of Bilberry Anthocyanins via Antioxidant, Anti-Inflammatory, and Anti-Apoptotic Mechanisms in a Visible Light-Induced Retinal Degeneration Model in Pigmented Rabbits

**DOI:** 10.3390/molecules201219785

**Published:** 2015-12-14

**Authors:** Yong Wang, Liang Zhao, Feng Lu, Xue Yang, Qianchun Deng, Baoping Ji, Fenghong Huang

**Affiliations:** 1 Beijing Key Laboratory of Functional Food from Plant Resources, College of Food Science & Nutritional Engineering, China Agricultural University, Beijing 100083, China; WangYongyffs@gmail.com (Y.W.); liangzhao@cau.edu.cn (L.Z.); 18201041642@163.com (F.L.); 13001994248@163.com (X.Y.); 2 Oil Crops Research Institute, Chinese Academy of Agricultural Sciences, Wuhan 430062, China; chunn2@163.com; 3 Hubei Key Laboratory of Lipid Chemistry and Nutrition, Wuhan 430062, China

**Keywords:** retinoprotection, bilberry anthocyanin, photoreceptor, light-induced retinal degeneration, pigmented rabbits

## Abstract

Excessive visible light exposure can induce damage to retinal cells and contribute to the development or progression of age-related macular degeneration. In this study we created a model of phototoxicity in pigmented rabbits. Furthermore, we investigated the protective effect of bilberry anthocyanin extract (BAE, Table A1) and explored the possible mechanisms of action in this model. The model of light-induced retinal damage was established by the pigmented rabbits exposed to light at 18,000 lx for 2 h, and they were sacrificed on day 7. After administration of BAE at dosages of 250 and 500 mg/kg/day, retinal dysfunction was significantly inhibited in terms of electroretinograms, and the decreased thicknesses of retinal outer nuclear layer and lengths of the outer segments of the photoreceptor cells were suppressed in rabbits with retinal degeneration. BAE attenuated the changes caused by light to certain apoptotic proteins (Bax, Bcl-2, and caspase-3). The extract increased the levels of superoxide dismutase, glutathione peroxidase, and catalase, as well as the total antioxidant capacity, but decreased the malondialdehyde level in the retinal cells. BAE inhibited the light-induced elevation in the levels of proinflammatory cytokines and angiogenic parameters (IL-1β and VEGF). Results showed that visible light-induced retinal degeneration model in pigmented rabbits was successfully established and BAE exhibited protective effects by increasing the antioxidant defense mechanisms, suppressing lipid peroxidation and proinflammatory cytokines, and inhibiting retinal cells apoptosis.

## 1. Introduction

Excessive visible light exposure can induce damage to retinal cells, especially to photoreceptor and retinal pigment epithelial (RPE) cells; such exposure may also contribute to the development or progression of age-related macular degeneration (AMD) and retinitis pigmentosa (RP) [1,2]. AMD is the leading cause of irreversible central vision loss in the elderly population in the world [3]. Prevalence data suggest that 1.75 million people are affected by AMD and 7 million people are at risk of developing AMD in the United States [4]. The pathogenesis of AMD is not well understood, but it is thought that a high oxygen concentration and high levels of polyunsaturated fatty acids in the retina, increasing sensitivity to light exposure and contribute to oxidative stress and inflammation, which injure the RPE, resulting in loss of photoreceptor cells [5,6].

Photochemical damage occurs after exposure to high energy radiation with a wavelength within the visible spectrum of light [7]. At the retinal level, exposure to light increases phagocytosis of the outer segment (OS) of photoreceptors and induces the formation of superoxide anions by RPE cells [8]. An imbalance develops between light-induced reactive oxygen species (ROS) and endogenous antioxidative systems, thereby resulting in photo-oxidative stress in the retina [9]. Additionally, impaired retinal antioxidant status leads to overexpression of proinflammatory and angiogenic parameters, which are primarily responsible for injured retinal microvasculature [10].

ROS expedite oxidative stress-induced damage of photoreceptor and RPE cells, and nutritional supplements such as polyphenol and xanthophyll that scavenge ROS can prevent or delay progression of early AMD [11,12]. Bilberries (*Vaccinium myrtillus* L.) are particularly rich in anthocyanins, such as delphinidin, malvidin, petunidin, cyanidin, and peonidin [13]. Various mechanisms of action have been proposed for anthocyanins, such as antioxidation, anti-inflammation, as well as effects on anti-apoptotic pathways and gene expression [14]. Anthocyanins have been shown to act directly as antioxidants to neutralize ROS by donating hydrogen ions and modulating cell signaling pathways [15]. A mouse model of endotoxin-induced retinal inflammation shows that an impairment in visual function, which has been improved after anthocyanin intervention [16]. Anthocyanins facilitate rhodopsin regeneration or interact directly with the rhodopsin molecule [17,18]. However, the possible mechanisms of protective effects of bilberry anthocyanins in visible light-induced retinal degeneration *in vivo* are still limited and controversial.

Pigmented rabbits were chosen because of melanin in RPE cells, which were similar to the structure of the human retina; moreover, their eye size is more accessible and allows for performing experimental operation [19]. In addition, fluorescent white light having an emission spectrum similar to daylight, which was chosen to mimic excessive exposure to daylight. In the present study, we generated a pigmented rabbit model of visible light-induced retinal damage that exhibits retinal cell oxidative stress, inflammation, angiogenesis, and apoptosis, which possesses several pathophysiological characteristics of AMD and can be used to investigate the mechanisms of retinal degeneration.

This study also aimed to elucidate the related mechanisms of the protective effects of bilberry anthocyanin extract (BAE) against visible light-induced retinal degeneration in this model. Scotopic electroretinogram (ERG), photopic ERG, and maximal response ERG (max-ERG) were performed at 1 day before and 1, 3, and 7 days after light exposure (18,000 lx for 2 h). Retinal damage was quantified by measuring the thickness of the outer nuclear layer (ONL) and OS length at 7 days after light-induced damage. Experiments tested the expression of apoptotic proteins (Bax, Bcl-2, and caspase-3) in retinal cells. We determined the changes in indices associated with oxidative stress metabolism, namely, superoxide dismutase (SOD), glutathione peroxidase (GSH-Px), catalase (CAT), total antioxidant capacity (TAOC), and malondialdehyde (MDA), in the retinal tissues. We also investigated the levels of proinflammatory cytokines (interleukin-1β (IL-1β)) and angiogenic parameters (vascular endothelial growth factor (VEGF)) in the retina.

## 2. Results and Discussion

### 2.1. Concentrations of Anthocyanins in Bilberry Anthocyanin Extract (BAE)

The anthocyanins in BAE were identified by LC-ESI-MS/MS. BAE contained 41.02% anthocyanins. The structure and concentrations of the 14 anthocyanins in the extract are shown in Table 1.

**Table 1:** Structure and concentrations of the individual anthocyanins in bilberry anthocyanin extract.
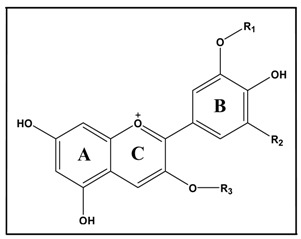

Identification	Structure			[M+] *m*/*z* Total/Aglycon	mg of ACN/g of BAE
	R1	R2	R3		
Delphinidin-3-galactoside	H	OH	Gal	465/303	36.67
Delphinidin-3 -glucoside	H	OH	Glu	465/303	44.92
Cyanidin-3-galactoside	H	H	Gal	449/287	43.36
Delphinidin-3-arabinoside	H	OH	Ara	435/303	54.60
Cyanidin-3-glucoside	H	H	Glu	449/287	52.30
Petunidin-3- galactoside	H	CH_3_O	Gal	479/317	15.42
Cyanidin-3-arabinoside	H	H	Ara	419/287	44.18
Petunidin-3-glucoside	H	CH_3_O	Glu	479/317	35.81
Peonidin-3-galactoside	CH_3_	H	Gal	463/301	7.14
Petunidin-3-arabinoside	H	CH_3_O	Ara	449/317	10.79
Peonidin-3 -glucoside	CH_3_	H	Glu	463/301	26.25
Malvidin-3-galactoside	CH_3_	CH_3_O	Gal	493/331	10.62
Malvidin-3-glucoside	CH_3_	CH_3_O	Glu	493/331	26.79
Malvidin-3-arabinoside	CH_3_	CH_3_O	Ara	463/331	1.31
Total anthocyanins					410.16

ACN: anthocyanin; BAE: bilberry anthocyanin extract; Glu: glucose; Gal: galactose; Ara: arabinose.

### 2.2. Effect of Visible Light Exposure on Retinal Function

**Figure 1:**
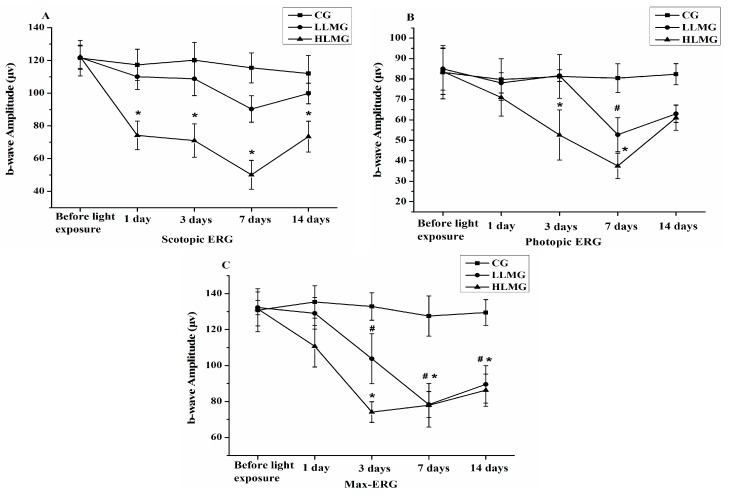
Values for the b-wave amplitudes of (**A**) scotopic; (**B**) photopic; and (**C**) max electroretinograms (ERGs) from retinas of rabbits in the CG, LLMG, and HLMG at 1 day before and 1, 3, 7, and 14 days after light exposure. Data are expressed as the mean ± standard deviation (*n* = 8). ^#^,* *p* < 0.05 (*t*-test). CG: no light exposure and vehicle administration; LLMG: 11,000 lx light exposure and vehicle administration; HLMG: 18,000 lx light exposure and vehicle administration.

Figure 1 shows that the b-wave amplitudes of scotopic ERG, photopic ERG, and max-ERG in the HLMG (high light-induced retinal damage model group: 18,000 lx light exposure and vehicle administration) on days 3 and 7 had significantly decreased compared with those in the CG (control group: no light exposure and vehicle administration, *p* < 0.05). While the b-wave amplitudes of scotopic ERG in the LLMG (low light-induced retinal damage model group: 11,000 lx light exposure and vehicle administration) were not significantly decreased compared with those in the CG (*p* > 0.05). Furthermore, the b-wave amplitudes of scotopic and photopic ERGs in the HLMG on day 7 were lower than those on days 1, 3, and 14 after exposure to light. Light reduced the b-wave amplitudes of scotopic ERG, photopic ERG, and max-ERG in the HLMG on day 7 by 56.59%, 53.46%, and 38.93%, respectively. Therefore, the model of light-induced retinal damage was established by the rabbits exposed to light at 18,000 lx for 2 h, and they were sacrificed on day 7 after the ERG was recorded.

Retinal diseases can be accurately detected by ERG, a technique that allows the quantitative and noninvasive assessment of retinal function [20]. Scotopic ERG and photopic ERG reflect the functions of rods and cones, respectively, and max-ERG reflects both rod- and cone-mediated activity [21,22]. The b-wave reflects principally a measure of photoreceptor, bipolar cell, and Muller cell functions [23]. The normal b-wave amplitudes of scotopic ERG, photopic ERG, and max-ERG were significantly attenuated by light exposure.

In exudative AMD, rods decline precipitously, while the photoreceptors that survive are largely cones [24]. The preferential vulnerability of rods over cones, consistent with the finding that the loss of scotopic sensitivity is greater than the loss of photopic sensitivity has been confirmed by visual functional status [24]. Also, this study was shown that the ERG data are consistent with the fact that the first to suffer are the rods at 1 day.

### 2.3. Protective Effect of Bilberry Anthocyanin Extract (BAE) on Visual Function

**Figure 2:**
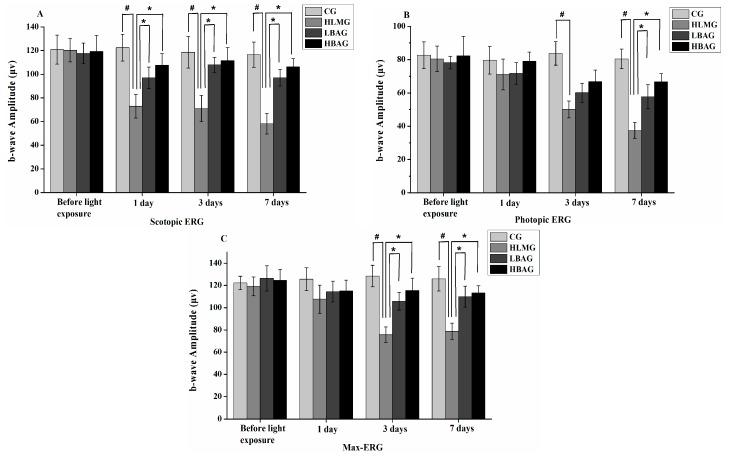
Values for the b-wave amplitudes of (**A**) scotopic; (**B**) photopic; and (**C**) max electroretinograms (ERGs) from retinas in rabbits in the CG, HLMG, LBAG, and HBAG at 1 day before and 1, 3, and 7 days after light exposure. Data are expressed as the mean ± standard deviation (*n* = 8). ^#,^* *p* < 0.05 (*t*-test). CG: no light exposure and vehicle administration; HLMG: 18,000 lx light exposure and vehicle administration; LBAG: 18,000 lx light exposure and administration of low dosage of bilberry anthocyanin extract (250 mg/kg/day); and HBAG: 18,000 lx light exposure and administration of high dosage of bilberry anthocyanin extract (500 mg/kg/day).

The reduction in scotopic ERG was significantly less in the LBAG (18,000 lx light exposure and administration of a low dosage of BAE group, 250 mg/kg/day) and HBAG (18,000 lx light exposure and administration of a high dosage of BAE group, 500 mg/kg/day) compared with that in the HLMG (*p* < 0.05) after 1, 3, and 7 days of light exposure. BAE (250 and 500 mg/kg/day) protected against the reduction of these amplitudes of scotopic ERG by 33.36% and 41.20% on day 7, respectively (Figure 2A). The reduction in photopic ERG was significantly less in the LBAG and HBAG compared with that in the HLMG (*p* < 0.05) after 7 days of light exposure. The extract (250 and 500 mg/kg/day) protected against the reduction of amplitudes of photopic ERG by 25.19% and 36.41% on day 7 (*p* < 0.05), respectively (Figure 2B). The reduction in max-ERG was significantly less in the LBAG and HBAG compared with that in the HLMG (*p* < 0.05) after 3 and 7 days of light exposure. BAE (250 and 500 mg/kg/day) protected against the reduction of these amplitudes of max-ERG by 24.72% and 27.33% at day 7, respectively (Figure 2C). Therefore, the reduction in the b-wave amplitudes of scotopic ERG, photopic ERG, and max-ERG was significantly less in the retinal degeneration rabbits after administration of BAE at dosages of 250 and 500 mg/kg/day. In the LBAG and HBAG, the ERG b-wave amplitude increased with increasing BAE concentrations.

### 2.4. Protective Effect of Bilberry Anthocyanin Extract (BAE) on the Outer Nuclear Layer (ONL) Thickness and Outer Segment (OS) Length

**Figure 3:**
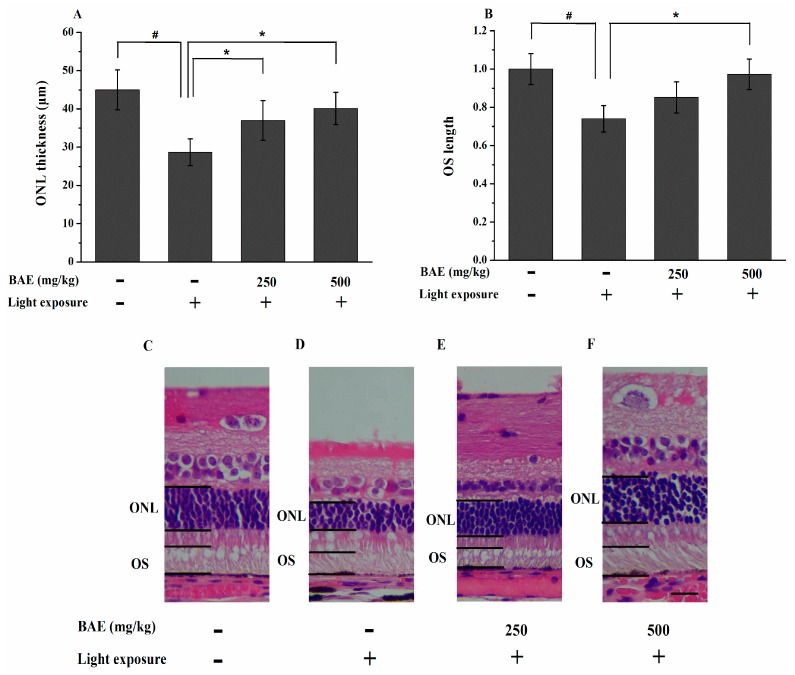
Effect of bilberry anthocyanin extract on the thickness of the outer nuclear layer (**A**) and the outer segment length (**B**) for the rabbit retina at 7 days after light exposure (18,000 lx) were examined by histological analysis. Thickness of the ONL were measured within 250–2750 μm (counted at every other 500 μm interval) of the superior and inferior edges to the optic nerve head (ONH) based on photographs of HE-stained sections. The OS length was measured 1 mm from the ONH in the superior and inferior retina. Data are expressed as the mean ± standard deviation (*n* = 8). ^#,^* *p* < 0.05 (*t*-test); (**C**–**F**) Representative images of the HE-stained sections of retinas of the rabbits. ONL: outer nuclear layer; OS: outer segment. Scale bar: 20 μm.

The effects of BAE on light-induced retinal damage were further examined by histological analysis at 7 days after light exposure. The thickness of the ONL in the LBAG and HBAG treated with 250 and 500 mg/kg/day BAE was significantly greater than that in the HLMG (36.99 ± 5.19 and 40.12 ± 4.20 μm *vs.* 28.67 ± 3.50 μm, respectively; *p* < 0.05) (Figure 3A). The HE-stained retinal sections showed that the OS length in the HBAG was increased by 23.18% compared with that in the HLMG (*p* < 0.05) (Figure 3B). Representative retinal images from all study groups are shown in Figure 3C–F.

Analysis of ONL thickness is an indicator of photoreceptor cell survival. Lipid peroxidation may be involved in the retinal damage that occurs following light exposure [25]. The damage may be too great and continues its course; thus, the progressive destruction leads to the greatest loss of ONL thickness observed after 7 day of light exposure. Our result is consistent with observations of Noell damage that occurs initially in the photoreceptors [26]. In addition, the current histological analysis clearly showed that light caused photoreceptor death, suggesting this phenomenon to be the major cause for the reduction in the b-wave amplitudes. In this study, the administration of BAE significantly suppressed light-induced photoreceptor degeneration. Previous studies demonstrated that light exposure triggers apoptosis in the ONL, whereas apoptosis is prevented by dietary antioxidants, such as lutein, astaxanthin, and anthocyanin [27,28,29].

Photoreceptor cells were mostly cleared from the retina and RPE cells were observed phagocytosing photoreceptor remnants after light exposure [1]. Blue light-exposed showed reduced OS length in mice indicating that the photoreceptor cells were damaged [30]. The ability of OS to manipulate O_2_ can better explain the pathogenesis of many retinal degenerative diseases ascribed to oxidative stress. Roehlecke *et al.*, demonstrated that blue light possibly induces ROS generation and oxidative stress in the OS of photoreceptors via NADPH oxidase as well as the mitochondria-like activity of the OS [31]. Recent data, showing that the rod OS expressed and activated cytochrome *c* oxidase and ATP synthase through extra-mitochondrial oxidative phosphorylation, to supply ATP for the visual transduction [32]. Calzia *et al.*, further reported that polyphenol dietary supplements regulated the F_o_F_1_-ATP synthase activity of the rod OS reducing production of ROS by the OS ectopic electron transport chain [33]. However, the reduction of OS length were prevented by suppressing the generation of ROS with the administration of BAE.

### 2.5. Bilberry Anthocyanin Extract (BAE) Protected Rabbits from Light-Induced Apoptosis in Retina

**Figure 4:**
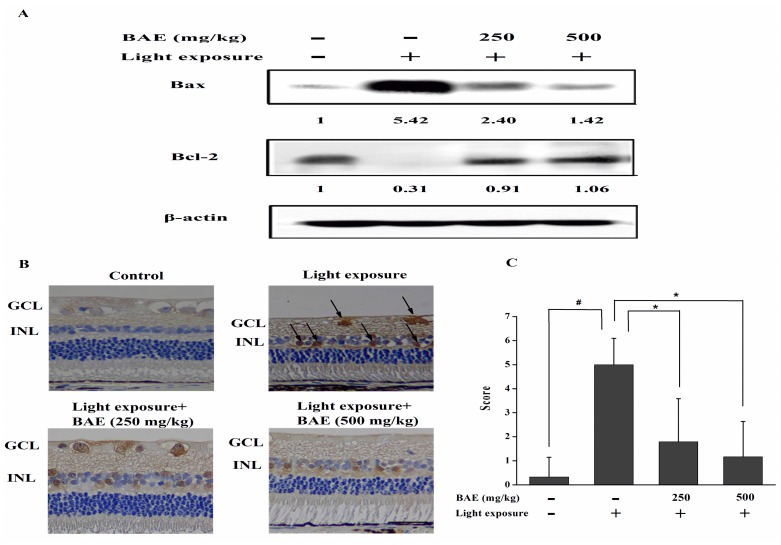
(**A**) Effect of bilberry anthocyanin extract on Bax and Bcl-2 expression in retinas based on Western blot analysis at 7 days after light exposure (18,000 lx). The data are representative of three independent experiments; (**B**) Active caspase-3 expression in retinas at 7 days after light exposure. INL: inner nuclear layer; GCL: ganglion cell layer. Scale bar: 20 μm; (**C**) Scores were determined by evaluating the extent and intensity of immunopositivity. Data are expressed as the mean ± standard deviation (*n* = 6). ^#,^* *p* < 0.05 (*t*-test).

The expression of apoptotic proteins were revealed by western blot analysis or immunohistochemistry. Light exposure resulted in the up-regulation of the pro-apoptotic protein Bax and down-regulation of the anti-apoptotic protein Bcl-2 (Figure 4A). Besides increasing the expression of the anti-apoptotic Bcl-2, BAE decreased the expressions of Bax. Caspase-3 activity was not detectable in the CG rabbits (Figure 4B). The expression of active caspase-3 was high in the inner nuclear layer and ganglion cell layer in rabbit retinas from the HLMG. However, treatment with BAE reduced the level of active caspase-3 in both layers. The expression of active caspase-3 by immunohistochemistry was scored as shown in Figure 4C. These findings indicated that activation of caspase cascade could be one of the mechanisms of light-induced apoptosis in this model and BAE could attenuate these changes.

In previous research, it was shown that exposure to 3000 lx of cool white fluorescent light for 2 h of albino rats caused photoreceptors and RPE cells apoptosis [34]. High-light intensity exposure leads to the photoreceptor apoptosis, which might due to the changes in mitochondrial membrane permeability [1]. Bcl-2 is directly regulated mitochondrial membrane permeability and integrity [35], and modulate the anti-oxidative capacity of cells [36]. Bax is known to influence the mitochondrial permeability transition pore, which has been proposed to be involved in the release of pro-apoptotic factors from mitochondria [1]. Several reports have indicated that the activation of caspase-3 may be one of the final executioners of photoreceptor cell death following light damage *in vivo* [37]. In the present study, BAE could attenuate the light-induced up-regulation of Bax and down-regulation of Bcl-2 in the retina, which involved the caspase machinery, by significantly reducing the expression of cleaved caspase-3. These effects indicated the neuroprotective action of the extract in light-induced retinal cell apoptosis.

### 2.6. The Activities of SOD, GSH-Px and CAT, Levels of MDA and TAOC

**Figure 5:**
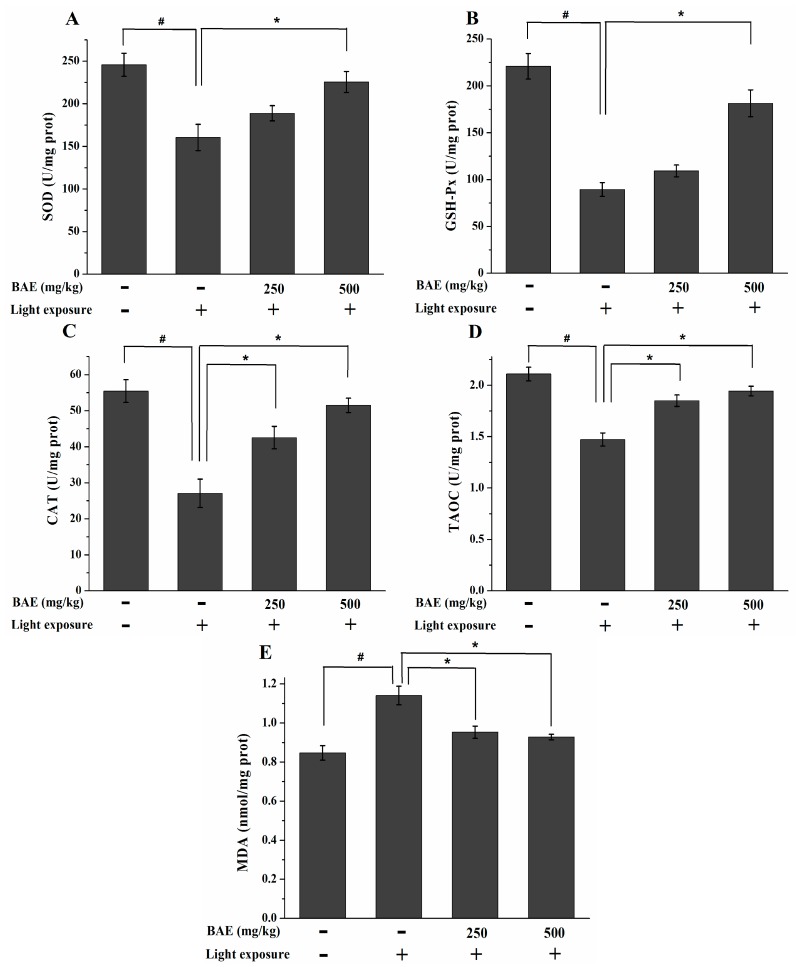
Changes in (**A**) SOD; (**B**) GSH-Px; and (**C**) CAT activities; as well as the (**D**) TAOC and (**E**) MDA levels, in the retina at 7 d after light exposure (18,000 lx). Data are expressed as the mean ± standard deviation (*n* = 8). ^#,^* *p* < 0.05 (*t*-test). SOD: superoxide dismutase; GSH-Px: glutathione peroxidase; CAT: catalase; TAOC: total antioxidant capacity; MDA: malondialdehyde.

At 7 days after exposure, the activities of SOD, GSH-Px and CAT were significantly lower in the HLMG than those in the CG (*p* < 0.05; Figure 5A–C). The activities of SOD, GSH-Px and CAT were significantly higher in the HBAG than those in the HLMG (*p* < 0.05). CAT activity was also significantly higher in the LBAG than that in the HLMG (*p* < 0.05). Rabbits in the HLMG showed a significant decrease in TAOC and a marked elevation in the MDA content (compared with the CG, *p* < 0.05; Figure 5D,E), which indicated a decline in antioxidant status. The rabbits treated with BAE in the LBAG and HBAG showed significantly increased TAOC by 18.01% and 22.27%, respectively (compared with the HLMG, *p* < 0.05). Compared with the HLMG rabbits, the MDA levels of the LBAG and HBAG rabbits significantly decreased by 22.35% and 24.71%, respectively (*p* < 0.05).

The endogenous antioxidant enzymes system can be activated after the light damage [38]. However, if illumination persists, excessive ROS production overcomes this defensive mechanism and causes oxidative stress that can damage the photoreceptors [39]. SOD, GSH-Px, and CAT can scavenge ROS in the body, but these endogenous antioxidative enzymes can be damaged by excessive ROS. BAE has been shown to increase the levels of SOD, GSH-Px, and CAT in retinal cells. The high levels of polyunsaturated fatty acids (PUFAs) in the photoreceptors increase susceptibility to lipid peroxidation. MDA, a degraded product of lipid peroxidation, is detected in this study [40]. The MDA levels in the LBAG and HBAG were significantly lower than those in the HLMG, whereas TAOC in the LBAG and HBAG were significantly higher than that in the HLMG. Therefore, BAE can reduce the consumption of antioxidative enzymes and decrease the levels of lipid peroxidation in the retina.

The retina is particularly susceptible to surrounding light damage, including high consumption of oxygen, large doses of visible light exposure, high proportion of PUFAs, and numerous chromophores [41]. Previous studies have reported that anthocyanins exert a protective effect on retinal cells after light-induced damage *in vitro* [8,42]. Our results suggested that BAE could increase the antioxidant defense mechanisms that counteract retinal oxidative stress.

Vitamin A aldehyde-conjugates form in photoreceptor OS membrane and are transferred to lysosomes of RPE cells when outer segment membrane is phagocytosed. These bisretinoid molecules such as all-trans-retinal dimer and A2E become the lipofuscin of RPE cells [43]. Bisretinoids are efficient producers of singlet oxygen when exposed to visible light [44]. The generation of singlet oxygen induces lipid peroxidation and the photo-oxidation of A2E and all-trans-retinal dimers. Cellular membrane damage, and DNA damage result and these events contribute to retinal cell death [45]. Anthocyanins are known for their catalytic capacity to detoxify singlet oxygen [8]. Therefore, the findings of this study indicated that singlet oxygen production by light irradiation may have been prevented by the existence of anthocyanins, such that oxidative stress was weakened and retinal cell death was reduced.

### 2.7. The IL-1β and VEGF Levels

**Figure 6:**
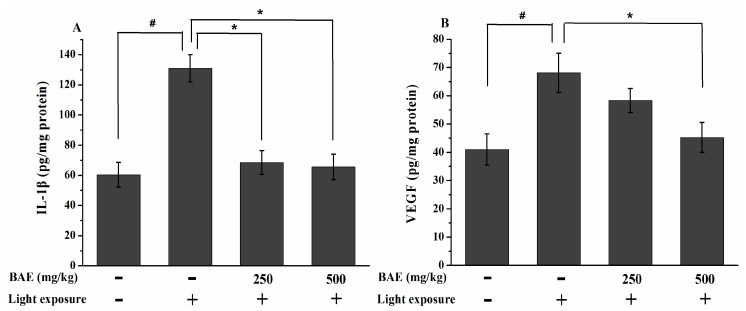
Changes in (**A**) IL-1β and (**B**) VEGF levels in the retina at 7 days after light exposure (18,000 lx). Data are expressed as the mean ± standard deviation (*n* = 8). ^#,^* *p* < 0.05 (*t*-test). IL-1β: interleukin-1 β; VEGF: vascular endothelial growth factor.

Visible light exposure resulted in marked increases in retinal IL-1β and VEGF concentrations in the HLMG compared with those in the CG (*p* < 0.05; Figure 6A,B). Compared with the HLMG, the LBAG and HBAG demonstrated significantly reduced IL-1β level (*p* < 0.05), and only HBAG exhibited markedly decreased VEGF (*p* < 0.05).

ROS is strong stimuli for the release of proinflammatory cytokines and angiogenic parameters, such as IL-1β and VEGF, which damage retinal cells and play an important role in the pathogenesis of retinal degeneration [46]. Consequently, oxidative stress exerted on the retina results in the activation of nuclear factor-κB (NF-κB), which is an inflammatory cascade, and acts as a trigger for the release of pro-inflammatory cytokines [47]. IL-1β is generated in RPE cells and choroidal neovascularization membranes. It is a proinflammatory cytokine that upregulates angiogenesis and induces neuroinflammation [48,49]. In addition, IL-1β is involved in the activation of several apoptosis regulatory genes [50]. VEGF is a cytokine with strong angiogenic and mitogenic actions; thus, it plays a major role in retinal vascular leakage, new choroidal blood vessel growth, neovascular AMD development, and neural retinal degeneration [51,52,53]. We provide data showing that BAE can attenuate those proinflammatory cytokines and angiogenic parameters which were affected by light damage.

This model mimics the retinal oxidative stress and focal photoreceptor and RPE degeneration found in AMD. Retinas from pigmented rabbits exposed to visible light (18,000 lx for 2 h) after 7 days showed a decrease in the b-wave amplitudes of the ERGs, retinal ONL thickness, and OS length, as well as higher levels of oxidative stress, lipid peroxidation, proinflammatory cytokines, angiogenic parameters, and apoptosis. Thus, a satisfactory light-induced retinal degeneration model was established. Furthermore, BAE significantly inhibited the light-induced reduction of the b-wave amplitudes of the ERGs and attenuated the effects of light damage to the retina upon histological examination. Biochemical data also showed that some apoptotic proteins, endogenous antioxidant enzymes, proinflammatory cytokines, and angiogenic parameters were affected by light damage but attenuated by BAE.

## 3. Experimental Section

### 3.1. Chemicals and Reagents

BAE purchased from JF-NATURAL Chemical Co. (Tianjin, China) was suspended in PBS at a concentration of 250 mg/mL. Methyl cyanide was of chromatographic grade and purchased from Merck (Darmstadt, Germany). Formic acid was purchased from Sigma (St. Louis, MO, USA). Deionized water was produced using a Milli-Q water-purification system (Millipore, Billerica, MA, USA). Tropicamide eye drops were purchased from Xingqi Pharmaceuticals Co., Ltd. (Shenyang, China). Sumianxin was purchased from Shengda Pharmaceuticals Co., Ltd. (Dunhua, China). Anti-Bax (ADI-AAS-040) was purchased from Enzo Life Sciences (New York, NY, USA). Anti-Bcl-2 (PAB19562) was purchased from Abnova (Taipei, Taiwan). Anti-caspase-3 (ab82585) was purchased from Abcam (Cambridge, UK). Anti-β-actin were purchased from Cell Signaling Technology (Danvers, MA, USA). RIPA buffer and bicinchoninic acid (BCA) protein assay kit were purchased from Beyotime Institute of Biotechnology (Shanghai, China). All other chemicals and reagents were purchased from Sigma-Aldrich.

### 3.2. Liquid Chromatography-Electrospray Ionization Tandem Mass Spectrometry (LC-ESI-MS/MS) Analysis of Bilberry Anthocyanin Extract (BAE)

LC-ESI-MS/MS analysis was performed using an Agilent 1260 series HPLC combined with an Agilent 6460 Series Triple Quad LC/MS equipped with a Jet Stream ESI source (Agilent Technologies, Santa Clara, CA, USA). MS was operated in the positive ion mode. Nitrogen was used as a collision gas. The analytical column was a 250 mm × 4.6 mm i.d. Agilent Zorbax SB-C18 column (Agilent, Palo Alto, CA, USA), which was maintained at 25 °C. Prior to analysis, all samples were filtered through a 0.45 μm membrane filter. The injection volume was 20 μL. The elution solvents, namely, (A) methyl cyanides with 2% formic acid and (B) H_2_O with 2% formic acid, were applied as follows: isocratic 3% A for 3 min, from 3% to 15% A for 12 min, from 15% to 25% A for 40 min, from 25% to 3% A for 3 min, and isocratic 3% A for 7 min. The flow rate was 0.5 mL/min, and detection was performed at 520 nm. The detailed MS conditions were as follows: collision energy, 15 eV; gas temperature, 300 °C; gas flow, 7 L/min; nebulizer pressure, 35 psi; sheath gas temperature, 300 °C; sheath gas flow, 11 L/min; capillary voltage, 3.5 kV; and nozzle voltage, 500 V.

### 3.3. Animal Care

A total of 40 healthy pigmented rabbits weighing 2.5–3.0 kg were purchased from the Animal Center of the Beijing Kaiyuan Co. (Beijing, China). All procedures were performed according to the Association for Research in Vision and Ophthalmology Statement for Use of Animals in Ophthalmic and Vision Research. The procedures were approved by the Ethical Committee for Animal Experimentation of the First Hospital Affiliated to General Hospital of the Chinese People’s Liberation Army. All rabbits were housed at a 12 h light-dark cycle for one week at 22–25 °C and 55%–60% humidity. All rabbits were freely fed a standard breeding diet (Beijing Ke Ao Xie Li Co., Beijing, China).

### 3.4. Treatment with Bilberry Anthocyanin Extract (BAE) and Exposure to Light

After a week-long adaptation period, the rabbits were randomly divided into five groups: control group (no light exposure and vehicle administration; CG) (*n* = 8), low light-induced retinal damage model group (11,000 lx light exposure and vehicle administration; LLMG) (*n* = 8), high light-induced retinal damage model group (18,000 lx light exposure and vehicle administration; HLMG) (*n* = 8), treatment group 1 (18,000 lx light exposure and administration of a low dosage of BAE group, 250 mg/kg/day; LBAG) (*n* = 8), and treatment group 2 (18,000 lx light exposure and administration of a high dosage of BAE group, 500 mg/kg/d; HBAG) (*n* = 8). The prescribed dosages of BAE were intragastrically administered to the rabbits. Administration began two weeks before light exposure and continued for one more week thereafter.

After dark adaptation (60–100 lx) for 24 h, the pupils were dilated with tropicamide eye drops at 20 min before light exposure. Non-anesthetized rabbits were then exposed to 11,000 ± 1000 or 18,000 ± 1000 lx (illumination meter: TES-1332A; TES Electrical Electronic Corporation, Taipei, Taiwan) of diffused cool-white fluorescent light (Zhongcheng, Guangdong, China) for 2 h in cages with reflective interiors (50 cm × 45 cm × 35 cm). The temperature during exposure to light was maintained at 25 ± 1.5 °C. After exposure to light, all the rabbits were placed in the dark for 24 h before they were returned to the normal light/dark cycle.

### 3.5. Electroretinograms (ERGs)

ERGs were recorded by a visual electrophysiology system (APS-2000AER, Kanghua Rui Ming Technology Co., Ltd., Chongqing, China) to measure retinal function. To allow the electrodes to stick to the rabbits’ skin, body hair on the forehead and bitemporal region was scraped off with a scalpel. ERGs were simultaneously recorded from both eyes by two golden-ring electrodes, two forehead reference electrodes, and one ground electrode in the bitemporal region. The standard ERG signals were recorded at 1 day before, as well as 1, 3, 7, and 14 days after light exposure, according to previously described methods [54]. After dark adaptation for more than 1 h, the rabbits were anesthetized with an intramuscular injection of sumianxin (0.2 mL/kg). Pupils were fully dilated with tropicamide eye drops. After anesthesia, methylcellulose ophthalmic solution was dripped on the left eye, and the right eye was covered with gauze and a black eyeshade. According to the standards set by the International Society for Clinical Electrophysiology of Vision, ERGs of the left eye were recorded before those of the right eye. All procedures were performed under dim red light.

### 3.6. Hematoxylin and Eosin (HE) Staining

After the ERG was recorded, the rabbits were sacrificed. After marking the 12 o’clock position of the cornea with a silk suture, the eyeballs were quickly enucleated and the eyeball was immersed for 48 h in a fixative solution containing 2.5% glutaraldehyde and 2% paraformaldehyde. Dehydration was achieved via successive baths of ethanol at increasing concentrations, followed by clearing with xylene in an automatic tissue processor. Samples were embedded in paraffin taking into account the sample orientation, and 4 μm slides were obtained with a microtome. Six paraffin-embedded sections were cut through the optic nerve head (ONH) of each eye. These sections were prepared with the standard procedure, stained with HE, and observed under a light microscope (Leica, Heidelberg, Germany).

### 3.7. Measurement of the Outer Nuclear Layer (ONL) Thickness and Outer Segment (OS) Length

For ONL thickness and OS length quantification, light micrographs were photographed. The ONL thicknesses were counted within 250–2750 μm (counted at every other 500 μm interval) of the superior and inferior edges to the ONH based on photographs of HE-stained sections by personnel blinded to the study groups. The mean ONL thickness was calculated from three sections (randomly selected from the six sections) for each retina. The OS length was measured 1 mm from the ONH in the superior and inferior retina (one eye of each of eight rabbits).

### 3.8. Western Blot Analysis

Whole-cell lysates from the retinal extracts were prepared for western blot analysis by sonication in RIPA buffer containing a protease inhibitor cocktail (Roche, Indianapolis, IN, USA), followed by centrifugation at 15,000× *g* for 30 min at 4 °C to collect the supernatant. After the protein concentrations were determined with a BCA protein assay kit, equal aliquots (20–30 μg) of protein samples were applied to 10% sodium dodecyl sulfate polyacrylamide gels (Invitrogen) and electrophoretically separated. Resolved proteins were electrophoretically transferred to nitrocellulose membrane (Millipore) and blocked with 5% nonfat dry milk for 1 h at room temperature. The membranes were incubated with Bax (1:1000), Bcl-2 (1:20), or β-actin (1:5000) antibodies for 2 h at room temperature, after which they were incubated with the appropriate horseradish peroxidase-conjugated secondary antibody for 2 h at room temperature. The signals were visualized by enhanced chemiluminescence (Fisher/Pierce, Rockford, IL, USA) and recorded on X-ray films (Estman Kodak Company, Rochester, NY, USA). The intensities of the protein bands were determined with ImageJ software (1.32j, National Institutes of Health, Bethesda, MD, USA).

### 3.9. Immunohistochemistry for Caspase-3

Sections of 4 μm thickness were prepared for immunohistological staining. Endogenous peroxidase was quenched by freshly prepared 3% H_2_O_2_ with 0.1% sodium azide. The treated sections were placed in antigen retrieval solution (0.01 mol/L citrate buffer, pH 6.0) for 15 min in a microwave oven at 100 °C and 600 W. The samples were blocked in 10% fetal bovine serum in PBS and incubated at 4 °C overnight in primary antibody solution of anti-caspase-3 (ab2171, 1:50). After washing with 0.01 M PBS buffer, the samples were incubated with horseradish peroxidase-conjugated secondary antibodies (1:200, Dako, Glostrup, Denmark) for 60 min at room temperature, developed with 3,3′-diaminobenzide tetrahydrochloride, counterstained with hematoxylin, dehydrated, and mounted. Consistent negative controls were obtained by replacing primary antibody with PBS. In case of discrepancies, a final score was established by reassessment on a double-headed microscope. During the scoring of caspase-3 expression, the extent and intensity of immunopositivity were both considered. The staining intensity was scored as follows: 0, negative; 1, weak; 2, moderate; and 3, strong. Positivity was quantified according to the percentage of positive cells: 0, <5%; 1, >5%–25%; 2, >25%–50%; 3, >50%–75%; 4, >75%. The final score was determined by multiplying the intensity and quantity scores, which yielded a range from 0 to 12.

### 3.10. Determination of the Activities of SOD, GSH-Px and CAT, Levels of MDA and TAOC

For biochemical analysis purposes, the retina was homogenized with an Ultra-Turrax apparatus (IKA T10basic; Staufen, Germany). The TAOC and MDA levels, as well as the activities of SOD, GSH-Px, and CAT, in the retinal homogenate were determined with assay kits purchased from the Nanjing Jiancheng Bioengineering Institute (Nanjing, China) following the manufacturer’s instructions. The protein concentrations were determined with the BCA protein assay kit.

### 3.11. Determination of the IL-1β and VEGF Levels

IL-1β and VEGF levels in retina were estimated using a commercially available ELISA kit from Keyingmei Biotechnology and Science Inc. (Beijing, China) following the manufacturer’s instructions. The protein concentrations were determined with the BCA protein assay kit.

### 3.12. Statistical Analysis

Values were expressed as the mean ± standard deviation. Data shown in the study were obtained from at least three independent experiments. Statistical comparisons between different groups were determined using one-way ANOVA followed by Tukey’s test. Values with *p* < 0.05 were regarded as significant.

## 4. Conclusions

The present study demonstrated that visible light-induced retinal degeneration model in pigmented rabbits was successfully established and BAE could protect retinal function and the histological integrity of the retina. This protective function was probably caused by the ability of bilberry anthocyanins to increase the antioxidant defense mechanisms, suppress proinflammatory cytokines, and inhibit retinal cells apoptosis. Therefore, bilberry anthocyanins are promising candidates as nutritional supplements for the prevention and inhibition of the progression of AMD and RP.

## Appendix

**Table A1:** Abbreviations.

Abbreviation	Original Word
BAE	Bilberry anthocyanin extract
RPE	Retinal pigment epithelial
RP	Retinitis pigmentosa
ERG	Electroretinogram
SOD	Superoxide dismutase
CAT	Catalase
MDA	Malondialdehyde
HLMG	High light-induced retinal damage model group
HBAG	High dosage of BAE group
ONL	Outer nuclear layer
HE	Hematoxylin and eosin
NF-κB	Nuclear factor-κB
VEGF	Vascular endothelial growth factor
AMD	Age-related macular degeneration
OS	Outer segment
max-ERG	Maximal response ERG
GSH-Px	Glutathione peroxidase
TAOC	Total antioxidant capacity
CG	Control group
LLMG	Low light-induced retinal damage model group
LBAG	Low dosage of BAE group
ONH	Optic nerve head
ROS	Reactive oxygen species
PUFAs	Polyunsaturated fatty acids
